# Case of Intestinal Obstruction by a Diverticulum Verum of Meckel

**Published:** 1878-07

**Authors:** 


Case of Intestinal Obstruction by a Diverticulum Verum of Meckel.—A male child, aged five years, had, since six months of age, been subject to repeated attacks of violent colic. During the last year of his life he had several attacks, which were treated by different physicians as verminous colic, by means of santonine, etc. The exhibition of this drug was constantly followed by the passage of lumbricoides. At other times no physician would be called; tr. opii. camph. would be given by the parents, to be followed at longer or shorter intervals by relief. These attacks always came on suddenly, while the child was in the midst of his play, and after periods of suffering, the pain would as suddenly subside. None of these attacks had been accompanied with vomiting, that I could learn, but he would be cool and bathed with sweat.

On Tuesday morning, April 16th, he was attacked with great violence. A physician was called, who, on learning the history of the previous attacks, diagnosticated worms, and gave santonine. Several powders were left, combined with sulphate of magnesia, to be repeated from time to time. All these doses were vomited. There was no remission of symptoms during the day.

On Wednesday morning the patient was again visited, and morphine given to quiet pain. The vomiting increased, and at six P.M., thirty-six hours after the attack, I saw him. He was sitting half erect in the bed, and could not lie down, because it increased the pain. The pupils were dilated, the features pinched, and he vomited soon after I entered the room, a dark, grumous fluid, looking like a diffuse blood-clot, only darker. The skin was cool, and the arms and hands cold to the elbows and cyanosed. The breathing was shallow and humid. The abdomen was distended and extremely tender on the right side of the umbilicus, and in this region a tumor could be made out on pressure, such as could be tolerated in this region. On the left side of the umbilicus there was scarcely any increased tenderness. After a careful review of the symptoms, I diagnosticated an internal strangulation to the right, and below the umbilicus. I did not think it could be intussusception, because there had been no discharge of blood and mucus from the bowels; and, second, the onset of the case and its progress had been much more rapid than is ordinary in such cases. I located the stricture in the small intestine at its lower portion, because the abdomen was greatly distended. The vomited matter was bloody and non-fecal. The abdomen would have been flat, and the vomited matter of a bilious character, if the stricture was high up. The absence of fecal matter showed the large intestine was not involved. Sometimes fecal matter is formed low down in the ileum, which would then cloud the diagnosis.

I directed the continuance of the morphine, and the use of injections, with small pieces of ice to relieve thirst. Prognosis, fatal. The child died at ten o'clock, P.M., four hours later.

Autopsey at 4 P.M., Thursday, eighteen hours after death. Present, Drs. T. W. Jones, N. R. Coleman and H. L. Agler, and George H. Colville, a medical student. *Rigor mortis* slightly marked, the abdomen beginning to be discolored from advancing decomposition. The abdomen was opened by a crucial incision. On reflecting the flaps, the bowels on the right side were almost black with scattered gangrenous patches, while those lying to the left of the median line showed all conditions, from a condition of health to the intensest congestion—as they lay near the umbilicus. A diverticulum arose from the right side of the umbilicus and one half an inch below, and was inserted into the ileum, about two feet from the ileo-cecal valve, opposite the insertion of the mesentery. Where the diverticulum was joined to the ileum there was a projection, of a cone shape, which was made up of all of the coats of the intestine, one inch in depth. To the point of this cone the ligament, which passed from the umbilicus, was inserted. This ligament was hollow for three-fourths of an inch, and on examination showed the remains of blood vessels therein. This latter circumstance showed that this ligature which caused the strangulation, was the remains of the omphalo-mesenteric artery of fetal life. Into a loop formed by this structure and the bowel, the intestines had passed from below upward. The loop was drawn tightly, and strangulation was complete. The calibre of the bowel above the stricture was greatly enlarged, while below it was small. The bowel was filled to a considerable extent with bloody matter, similar to what the child had vomited. The omentum did not enter into the stricture, for it was so short it did not reach so low.

As an incidental circumstance, I may mention that as a section of the intestine was made to remove the specimen, several dead lumbricoides escaped.

*Remarks.*—1. The autopsy accounted for the frequently recurring colics of this child, on the supposition that slight strangulation would occur at times which would be reduced by the movements of the bowels.

2. It revealed the cause of strangulation, which placed in its most favorable light the value of the operation of laparotomy. The diagnosis was complete, as the *post-mortem* showed, and an earlier recognition would have given the only chance for relief by an operation. The division of a bridle not larger than a crow quill, would have relieved the stricture, without any annoying search.

3. It placed in clear relief the utter hoplessness of all medical means.—*Ohio Medical Recorder.*

